# Implementation of Active Workstations in University Libraries—A Comparison of Portable Pedal Exercise Machines and Standing Desks

**DOI:** 10.3390/ijerph15061242

**Published:** 2018-06-12

**Authors:** Camille Bastien Tardif, Maude Cantin, Sylvain Sénécal, Pierre-Majorique Léger, Élise Labonté-Lemoyne, Mickael Begon, Marie-Eve Mathieu

**Affiliations:** 1 École de kinesiologie et des sciences de l’activité physique, Université de Montréal, Montreal, QC H3T 1J4, Canada; camille.bastien.tardif@umontreal.ca (C.B.T.); maudecantin@hotmail.com (M.C.); mickael.begon@umontreal.ca (M.B.); 2 HEC Montréal, Montréal, QC H3T 2A7, Canada; sylvain.senecal@hec.ca (S.S.); pierre-majorique.leger@hec.ca (P.-M.L.); elise.labonte-lemoyne@hec.ca (É.L.-L.); 3 Centre de recherche du Centre Hospitalier Universitaire Sainte-Justine, Montréal, QC H3T 1C5, Canada

**Keywords:** sedentary behaviors, prolonged sitting time, active workstations, standing workstations, university libraries, physical activity

## Abstract

Sedentary behaviors are an important issue worldwide, as prolonged sitting time has been associated with health problems. Recently, active workstations have been developed as a strategy to counteract sedentary behaviors. The present study examined the rationale and perceptions of university students’ and staff following their first use of an active workstation in library settings. Ninety-nine volunteers completed a self-administered questionnaire after using a portable pedal exercise machine (PPEM) or a standing desk (SD). Computer tasks were performed on the SD (*p* = 0.001) and paperwork tasks on a PPEM (*p* = 0.037) to a larger extent. Men preferred the SD and women chose the PPEM (*p* = 0.037). The appreciation of the PPEM was revealed to be higher than for the SD, due to its higher scores for effective, useful, functional, convenient, and comfortable dimensions. Younger participants (<25 years of age) found the active workstation more pleasant to use than older participants, and participants who spent between 4 to 8 h per day in a seated position found active workstations were more effective and convenient than participants sitting fewer than 4 h per day. The results of this study are a preliminary step to better understanding the feasibility and acceptability of active workstations on university campuses.

## 1. Introduction

Physical activity guidelines recommend at least 150 min of moderate to vigorous intensity aerobic physical activity per week for adults [1]. Nevertheless, only 32% of Canadians 18 to 39 years of age follows these recommendations [2]. This lifestyle is known to be directly linked to major chronic health problems, such as cardiovascular diseases, diabetes, cancers, obesity, hypertension, atherosclerosis, and osteoporosis [3]. In addition, physical inactivity can easily coexist with a sedentary lifestyle, known for its direct effect on an individual’s metabolic health [4]. Adverse consequences of being seated most of the day are also independent of those related to physical inactivity [5]. Prolonged sedentariness of postural muscles induces negative effects on cellular processes in skeletal muscles, such as regulation of triglycerides and cholesterol [5]. Globally, the incidence and the mortality related to cardiovascular disease, cancer, and type 2 diabetes are increased with high sedentary time [6], and it is estimated that the risk of all-cause mortality increases by 5% for each hour increment of daily sitting for adults who sit seven hours or more per day [3].

Research has shown that 55% of university students do not meet current physical activity guidelines [7], a phenomenon that starts in childhood [8]. In addition, students spent an average of eight hours per day sitting, either to study, watch television, do computer work, or relax [9]. Furthermore, 83% of university students reported spending 100% of their time sitting in class [7].

In order to intervene with this population, university libraries have been identified as an ideal venue, since they are preferred by students for academic activities [10], and thus, offer a good opportunity to reduce sedentary occupational time for students. In the library, student activities are predominantly sedentary, such as studying, reading, or using the computer [11]. In order to reduce sedentary behaviors, many approaches focus on encouraging people to exercise during leisure time. However, this approach targets improving physical activity rather than breaking up extended periods of sitting time. Promoting worksite physical activity interventions have also taken many approaches to reduce prolonged sitting time, but long-term results have been inconclusive [12]. Low intensity physical activity more integrated into work or school/study main activity may be a more direct and successful approach [13].

Recently, there has been interest towards active workstations, with a growing focus on reducing occupational sedentary time [14]. Active desks are designed to combine sedentary work and low intensity physical activity. There is evidence that suggests that there are improved health benefits, compared to simply increasing physical activity outside of sedentary time, of using low intensity activities maintained for longer periods of time [15]. The concept of active workstations has been introduced to increase the overall daily time of physical activity, while simultaneously decreasing the amount of inactive sitting time [16]. Previous research has shown that alternating between sitting and standing positions in university students could reduce sedentary time by 30 to 120 min per day [7]. More importantly, students participating in the study saw improvements in their physical health, attention span, and stress levels [7]. Another study showed that frequent postural changes throughout extended periods of sedentary time could lead to reductions in waist circumference [4]. As such, active workstations are of growing interest for metabolic health by reducing sedentary time and improving physical health parameters, such as reductions in body mass index (BMI) and muscular discomfort of the lower limbs [17].

Preliminary findings suggest that users are in favor of using active workstations, such as cycling and standing, and their implementation at work seems feasible [18]. A study by Benzo and colleagues [7] showed that students and instructors were both in favor of introducing standing desks to the classroom, and that students understood the benefits of introducing such stations. When given the opportunity, more than a half of the students (61%) responded that they would spend 25% to 50% of class time standing. Positive attitudes toward the use of pedal machines in a library were also shown [11]. However, concerns have been raised about the perceptions of different active workstations by university students, and about the implementation of such stations on campus [9].

Implementation of active workstations in university facilities could be beneficial to student health, especially since research clearly demonstrates that students are interested in such projects [7]. As a result, the objective of this study is to explore university student and staff views, usability, and feasibility concerning their initial use of different active workstations (portable pedal exercise machine or standing) in libraries, and recording the use and perceptions that they make of such workstations. The response to their first use was specifically studied given that active workstations were introduced for the first time on campus.

## 2. Materials and Methods

### 2.1. Recruitment and Participant Information

Recruitment and data collection was conducted at the Université de Montréal and the HEC Montreal campuses, where library visitors were mainly students and university employees. The publicity was conducted using our research team website and its social media platforms, in addition to gaining participants who voluntarily participated as they passed by the workstations. For the purposes of this study, only those who responded that it was their first use of an active workstation and that they noticed the presence of active desks were eligible to participate in the study.

This study was approved by the Research Ethics Boards of both institutions (ethical approval number: 16-167-CERES-D).

### 2.2. Experimental Setup

**Figure 1:**
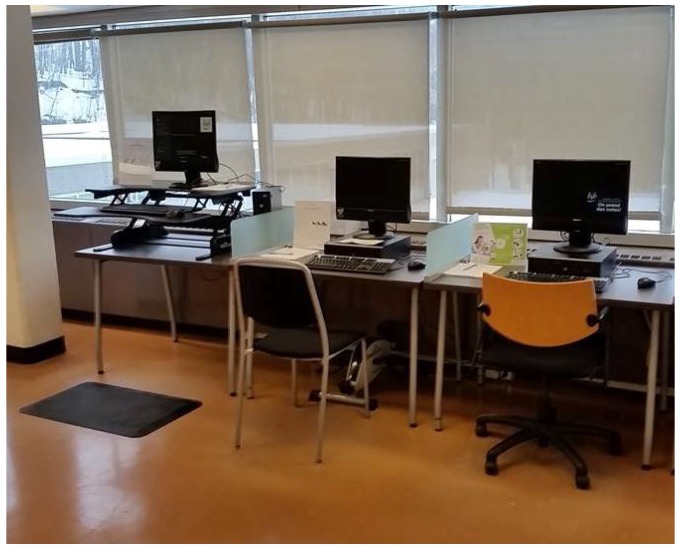
Setup of workstations in one of the three libraries (Kinesiology, Université de Montréal).

Three different workstations were set up side-by-side in a central position in three libraries of our campus: a conventional sitting desk (CSD), a portable pedal exercise machine (PPEM), and a standing desk (SD) (Figure 1). These stations were added to the natural environment of the libraries (i.e., materials from the libraries were used in this study). In two of the libraries, students could use their own laptops, while computers were provided for the third one.

### 2.3. Materials

**Figure 2:**
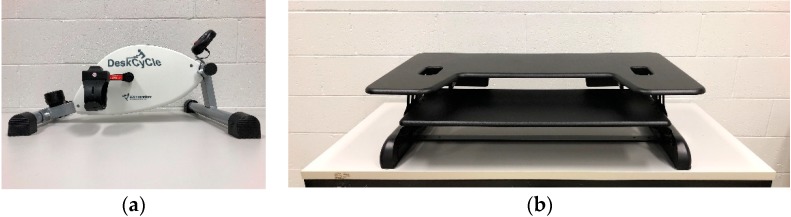
(**a**) Portable pedal exercise machine; (**b**) standing desk.

The first active workstation was a PPEM (DeskCycle^TM^, 3D Innovations, Greeley, CO, USA; Figure 2a). Participants were free to adjust the distance between themselves and the PPEM, and to set the pedal’s resistance. The second station used, SD, was the ProPlus 36^TM^ adjustable-height standing desk (Varidesk, Coppell, TX, USA; Figure 2b). On this SD, participants were able to adjust the height of the station, and an adjustable anti-fatigue mat that cushions and supports feet, knees, hips, and back was already installed. Finally, a CSD was set up. Instructions on how to use the workstations were displayed next to each station.

### 2.4. Protocol and Procedures

After each use, participants were invited to complete a self-administered questionnaire about their use and perceptions of their experience with the station. As compensation for their time, participants who completed a questionnaire were entered in to win one of the active workstations. Once completed, participants placed their questionnaires in a collection box in each library. For the current study, only questionnaires from a first use of the active workstations were analyzed. The active workstations and questionnaires were available at all times, with no time limit during library hours. Questionnaires were collected every week in all three libraries between March 2017 and June 2017.

### 2.5. Operationalization of Variables

All participants visiting the libraries were invited to complete the questionnaire on a voluntary basis. First, participants were required to identify the type of workstation used, and whether it was their first-time use. They also were asked to record their arrival and departure times in order to measure total utilization time. Next, participants were asked to indicate the use they made of the active workstations using a checklist: type of activity they were performing while using the active workstation, such as writing on paper, reading documents in paper format, reading electronic documents, emails, internet browsing, social media, Word, Excel, and PowerPoint or equivalents, and the type of material they used: pencil/paper, fixed computer, laptop, phone, or other means.

The questionnaire also included a nine semantic differential response measurement scale to assess user appreciation of the workstation. The items were adapted from Voss et al. [19] and used the Likert scale (1 to 7): (1) effective to ineffective, (2) useful to useless, (3) functional to not functional, (4) necessary to unnecessary, (5) convenient to inconvenient, (6) unpleasant to pleasant, (7) dull to exciting, (8) enjoyable to unenjoyable, and (9), comfortable to uncomfortable); these represent the utilitarian (#1–4) and hedonic (#6–9) dimensions of the participants’ appreciation. Users also replied to qualitative questions about the reason why they had chosen to use the workstations and any barriers they had encountered while using them.

Additional scales inspired by Tobin et al. [20] used Likert scales from 1 to 7 to determinate the desirability of such stations: (1) Whether participants were in favor of individuals using an active workstation; (2) Whether they felt like it was socially acceptable to use an active workstation; (3) Whether they intended to use more active workstations; and (4) if they felt equipped to use an active workstation. Moreover, the same scales were used as indicators of the probability and possibility of (1) that the participant would use an active workstation in the future at the library, and (2) that the participant would use an active workstation in the future elsewhere. The questionnaire also included a question about whether the presence of an active workstation had changed the participant’s intention to use the workstation. If the answer was “yes”, respondents were invited to specify to what degree they would be ready to pay for an active workstation.

Finally, to conclude the questionnaire, questions about participant characteristics were asked (age, height, body weight, and sex). The average daily sitting time and moderate-to-vigorous physical activity time for an average week, with questions adapted from the International Physical Activity Questionnaire [21], were also reported, to determine their habits regarding activity lifestyle.

### 2.6. Statistical Analysis

Values are mean ± standard deviation, unless otherwise specified. It is of note that the CSD stations were excluded from the analysis because of the small sample size (*n* = 21). To highlight differences between active workstations, chi-square tests were used for categorical variables and analysis of variance were used with numerical variables. Effect sizes calculated using pooled standard deviations are also presented and interpreted as small (0.1), medium (0.3), and large (0.5). Assumptions to the use of these tests were respected. In all cases, a statistical significance of 5% and a confidence interval of 95% were used. Analyses were performed using SPSS 24 (IBM, Armonk, New York, USA).

## 3. Results

### 3.1. Descriptive Analysis

Of 99 analyzed questionnaires, 43 were completed by males, 51 by females, and 5 participants did not specify their gender. This response rate represents less than 1% of library users. On average, participants (students and university employees) were 28.3 ± 9.8 years of age, and classified as normal weight (mean BMI 23.6 ± 4.6 kg/m^2^), self-reported sitting time averaged 7.7 ± 2.7 h per day, and practicing physical activity, an average of 4.1 ± 3.8 h per week (63% do a minimum of 150 min of moderate-to-vigorous physical activity per week). Mean duration of use was 50.9 ± 63.2 and 68.8 ± 98.7 min for PPEM and SD, respectively. These profiles were similar between users of each active workstation (*p* > 0.05).

### 3.2. Influence of User Characteristic on Station Choice and on Perceptive Appreciation of Active Workstations

Regarding the influence of user characteristics on station choice, only gender was shown to be significant. Differences in results were observed between gender and the preference of active workstations: females preferred PPEM over standing (65.2% vs 43.8%) while males preferred SD over PPEM (56.3% vs 34.8%) (*p* = 0.037). User characteristics had an effect on user perceptions and intentions. Significant results for the utilitarian and hedonic dimensions of participant appreciation of the active workstation were found. In individuals aged <25 years of age, the pleasure dimension (mean score of 2.25 ± 1.51; reverse scoring) was greater compared to individuals aged 26 to 40 years of age (mean score of 2.93 ± 2.02), and those aged 41 and over (mean score of 3.75 ± 1.91) (*p* = 0.020). Both SD and PPEM groups preferred 4 to 8 h of active desk use.

### 3.3. Influence of Station Choice

Significant differences were observed between the type of tasks accomplished on each active workstation (Table 1). Computer tasks (i.e., Office Suite) were performed 33.2% more often on the SD active workstation than on the PPEM (*p* = 0.001), and paperwork was performed 20.9% more often using the PPEM active workstation than on the SD workstation (*p* = 0.037). To be more specific, SD was more often used for Microsoft Word tasks (*p* = 0.006) than for reading paper documents (*p* = 0.048) than PPEM. Participants also reported that the use of laptops while operating SD stations was preferred over PPEM (*p* = 0.003) (Table 1).

No significant differences in self-reported desirability and intention to use an active workstation were observed, and effect sizes were small (Table 2).

**Figure 3:**
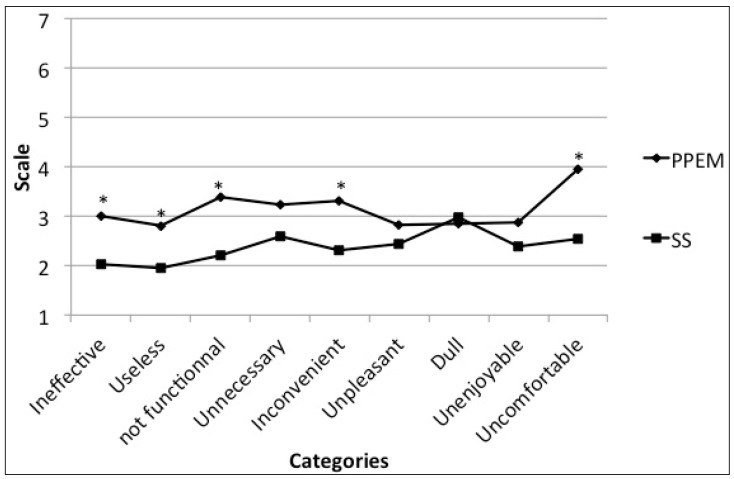
Appreciation of active workstations. Note: Responses were reported on a 1 to 7 scale, where 1 = very favorable and 7 = not favorable at all.

Globally, SDs were more acceptable than PPEMs (5 of 9 indicators, with remaining elements being equal; Figure 3). Four utilitarian dimensions and one hedonic dimension of participant appreciation revealed differences. A favorable appreciation of the SD active workstation (*p* < 0.001), with a 1.43 difference between the stations, was shown. No results over 4/7 were reported in all categories for both active workstations.

### 3.4. Qualitative Feedback from Users

Based on their qualitative responses and perceptions, some participants found significant productivity and health benefits while using active workstations:

“*This change of position for studying allows me to study longer*.” (SD)
“*Having pedals set in class might be relevant and could prevent me to draw on my notes or sleep when I can’t listen anymore. Also, it relaxes me*.” (PPEM)
“*It’s better for health, I have a hyperactive behavior and I have difficulty to concentrate. It’s hard for me to stay seated*.” (SD)
“*I was a little anxious, so I decided to try the station to see if it can help reducing my anxiety*.” (PPEM)
“(active workstation) *stimulated me in my studies.*” (PPEM)

On the other hand, some participants complained about difficulties in adjusting the stations:

“*Not enough space, chair poorly adjusted*.” (PPEM)
“*My legs touched the desk every time*.” (PPEM)
“*It’s hard to concentrate on studying and on the physical action at the same time*.” (PPEM)
“*Tired of remaining in a standing position*.” (SD)
“*Not stable enough. It’s moving when I write on it and the station is not high enough for me. I’m 1 m 80 tall*.” (SD)

Participants reported that their intention to use an active workstation was positive following their initiation, but not significantly different from one station to the other: 51.1% of PPEM users and 64% of SD (*p* > 0.05).

## 4. Discussion

This study was conducted with the objective of learning how university students and staff initially use and react to active workstations introduced in libraries. The results of this investigation suggest (1) a difference in station preference by gender, (2) a selection of some specific tasks according to the station type being used, (3) a difference in appreciation between the PPEM and the SD, and (4) some positive influence on library user opinions of the active desk as a result of the choice to use an active workstation and user characteristics.

In the current study, active individuals are interested in active workstations. Also, an equal proportion of men and women took part in the experiment, but men selected the SD to a greater extent than women, who preferred PPEM. A Maeda study [11] previously demonstrated that women had better participation in active workstation studies, suggesting that our setting might be well suited to both genders. According to a study by Le [22], males globally tended to have more postural transitions than females while using a workstation: seated, perching, and standing stations. That study confirms our own findings, since it was the SD which has fewer restrictions of motion that men prefer. Additionally, women have been known to be more committed to sedentary behaviors, such as sitting, than men [23]. Speculation about women who identified better with the PPEM station could be another reason for this preference.

The average amount of time spent in a sitting position in our sample, 7 h and 42 min, is consistent with previous studies. The time spent in overall sedentary behaviors in the United States is estimated to be 7.7 h/day, or 55% of waking hours [23]. More specifically, young adults between 20 and 29 years of age spent 7 h and 48 min a day performing sedentary behaviors [23]. These findings support our results: the need for interventions that reduce time spent in sedentary behavior, and that the active workstations installed in libraries better suited the interests of individuals requiring sedentary reduction interventions. Carr et al. [13] stated that, when used on a regular basis with an average of intensity 5/10 on a Borg scale, 23 min per day of PPEM could potentially result in health benefits. The participants in our study self-reported spending an average of 50.9 min per utilization, suggesting that students could benefit from PPEM’S positive side effects, such as increased energy expenditure.

Despite the fact that only a few participants accomplished reading tasks on active workstations, reading hard copy paper documents while using a PPEM was favored by participants. Previous investigations regarding the impact of cycling stations on reading comprehension found that PPEM and reading are two compatible tasks: they showed no negative effects on reading comprehension time or accuracy while using a cycling station [24]. The same results were noted while using a PPEM [25], supporting our analysis that suggests that reading a paper was more popular at PPEM than SD.

In the current study, computerized tasks were 33.2% more popular among SD users than PPEM users. More precisely, the use of Office Word was 27.5% more popular at SD than PPEM (Table 2). A study by Straker et al. [18] showed no difference between standing and sitting desks regarding typing, use of a mouse, and perceived performance. Furthermore, no reduction in performance was identified for standing desk users. However, the cycling desk did not show the same results. Actual and perceived typing, as well as mouse pointing performances, were, however, affected: 0.7% and 61% increases in error rates were observed, respectively. Moreover, a reduction in typing abilities was also observed. Interestingly, a study by Cho et al. [26] revealed that typing performance while using a PPEM differed according to the speed used. High speed pedaling resulted in the worst impact on typing abilities, compared to no pedaling and low-level pedaling conditions, which showed no significant differences between performances. The evidence suggests that the arm may have difficulty remaining independent during leg-cycling movements [16]. An interference of upper body motions and arm stability while the legs are moving in a circular fashion could have impacted performance [25]. This suggests that fine motor skills required by the upper body limbs, such as mouse pointing, may not be recommended, while the lower limbs perform gross motor skills, such as cycling [24]. Starker et al. [18] also demonstrated that combined keyboard and mouse tasking speeds were reduced during cycling. When coordination and delicate motor skills were required, the interdependence between the legs and the arms could also have reduced task performance [16]. This supports the results of our study, since laptops were used 29.8% more often at SD, knowing that a mouse or touchpad used with laptops requires more precise motor skills. According to Winter [27], it is best to use active static workstations for mobile tasks, and a dynamic workstation is more suitable for static tasks.

Globally, and related to their appreciation, participants had a positive experience and were in favor of using an active workstation. Carr et al. [13] showed similar results for pedal machines, and Benzo et al. [7] confirmed our findings for the SD. However, in our study, the SD was preferred by participants over the PPEM. According to Carr et al. [13] and the results presented above, the difficulty in performing computer tasks while using a PPEM could be the reason why SD was preferred. Additionally, the Maeda study [11] supported these findings, suggesting that participants were neutral regarding the effectiveness of studying or working normally while using a PPEM. Additionally, the SD was reported as more comfortable than the PPEM workstation. According to the Maeda study [11], participants neither agreed or disagreed about the comfort of a pedal machine, which supports our results that the average appreciation of the PPEM comfort seems neutral (3.97 on the Likert scale of discomfort), and that the SD was more comfortable, with a 2.53 Likert scale score of discomfort.

As to the desirability of active workstations, a study by Maeda [11] suggested that participants had a neutral attitude toward the presence of a pedal machine in a library for future use, which differs from our study results in which participants were mainly in favor. Furthermore, the Carr et al. study [13] demonstrated that participants seemed to have a balanced opinion regarding the social acceptability of using an active workstation in a public place. According to another study by Carr et al. [28] with a CD, the convenient aspect of the active workstation could potentially impact the use of active workstations. However, these findings do not support our study, where participants felt that it was socially acceptable to use an active workstation in a library. As to the intention of using the PPEM more often, and to use it away from a library or work environments, participants in the current study were in favor, similarly to the participants of the Carr et al. study [13].

Moreover, some participants complained of back pain while using the active workstations. Studies have demonstrated that prolonged standing time can cause musculoskeletal discomfort [18], and that inadequate standing increases intradiscal pressure, which can provoke pain [29]. The most significant report of discomfort and spinal loads with the most overall motion was attributed to a standing position compared to seated and cycling desks. In addition, the pain encountered might have been reinforced by more motion due to the discomfort of the desk not being properly adjusted [22]. Ergonomic factors and anthropometric measures should also be considered for the use of a PPEM, even if the use of such a station has not been shown to increase back pain [13]. Cho et al. [26] showed that a standard desk should not be used with this type of dynamic workstation. These ergonomic factors could improve the experience of PPEM, and reduce limitations for this station’s use. In the long term, safety, health, and wellbeing should be improved as well. Nevertheless, ergonomics remain a challenge in settings such as a library, where numerous individuals use a desk daily.

Similarly, and based on their qualitative responses and perceptions, some participants complained about the difficulty in adjusting the desks. They reported that their knees were bumping against the lower working surface when they used the PPEM. This same limitation was also shown in a study by Carr et al. [28]. Other studies have pointed out that the PPEM was not steady on the ground, thereby inhibiting participant abilities to cycle. However, these findings differ from the Carr study, which suggested that overall, participants found the pedal machine easy to use. For the SD, participants reported that it was difficult to adjust the desk to their height; more specifically, in taller participants. However, some participants also found significant health benefits with active workstations, such as helping reduce the symptoms of hyperactivity and anxiety during their use. A study by Silter [16] also suggested that physical activity was related to higher levels of wellbeing and ability to study.

In order to place this study in context, a few limitations must be noted. The introduction of laptops could be used in only two of the three libraries; the Health and the HEC-Montréal libraries and the PPEM used traditional desks. The study also provides a partial picture, since it does not provide any information on non-responder and non-users. Utilization beyond the first encounter also needs to be addressed. Despite these limitations, this study was conducted in the natural setting of libraries, where sedentariness is omnipresent. It reveals that students and employees are interested in trying active desks, at least for a portion of the day, and that a diversity of active desks is potentially important to consider, due to the fact that tasks performed differed between active desks, and that reaching less active individuals and maintaining interest will need specific attention.

## 5. Conclusions

Overall, this study suggests that the first use of active workstations in a library was positively appreciated by participants, and that the use that they made of them differed between the PPEM and the SD. The presence of active workstations in the library positively influenced more than 50% of individuals to use them in the future; in addition, station choice was influenced by sex, age, and time spent in a seated position per day. These findings encourage public health agencies to reduce sedentary time, and explore new innovative strategies to increase PA in university students. Further research is required to better understand the need of the effectiveness of such stations (PPEM and SD) in university libraries, and how they can be implemented in a more wide-ranging way.

## Figures and Tables

**Table 1:** Tasks made by the participants according to the active workstation used.

Tasks	PPEM	SD	Significant Level
PAPER	26 (54.2)	17 (33.3)	0.037
Handwriting	15 (31.3)	16 (31.4)	0.581
Read documents	18 (37.5)	10 (19.6)	0.048
ELECTRONIC	28 (58.3)	33 (64.7)	0.515
Read documents	19 (39.6)	22 (43.1)	0.720
Email	9 (18.8)	15 (29.4)	0.216
Internet (web)	20 (41.7)	20 (39.2)	0.804
Social media	4 (8.3)	6 (11.8)	0.571
OFFICE SUITE	17 (35.4)	35 (68.6)	0.001
Word or equivalent	15 (31.3)	30 (58.8)	0.006
Excel or equivalent	4 (8.3)	5 (9.8)	0.799
PowerPoint or equivalent	4 (8.3)	7 (13.7)	0.394

Portable pedal exercise machine (PPEM); standing desk (SD). Values are *n* (%).

**Table 2:** Future intent to use an active workstation.

Question	PPEM	SD	*p*-Value	Effect Size
Are you in favor of active workstations?	6.5 (1.4) 6.1–6.9	6.6 (1.0) 6.3–6.9	0.664	0.07
Do you feel that it is socially accepted to use an active workstation?	6.0 (1.5) 5.6–6.5	5.7 (1.5) 5.2–6.1	0.245	0.15
Do you have the intention to use an active workstation more often?	6.0 (1.7) 5.5–6.4	6.4 (1.1) 6.1–6.7	0.155	0.05
Do you feel well-enough trained to use an active workstation?	5.0 (2.3) 4.3–5.6	5.2 (2.0) 4.6–5.7	0.636	0.05
Do you plan to use an active workstation at the library in the future?	6.0 (1.8) 5.5–6.5	6.3 (1.2) 6.0–6.6	0.261	0.15
Do you plan to use an active workstation somewhere else in the future?	5.3 (2.1) 4.7–5.9	5.8 (1.5) 5.4–6.3	0.141	0.17
Did the presence of active workstations in the library change your intention to use an active station?			0.228	
Yes, *n* (%)	24 (51.1)	32 (64.0)		
No, *n* (%)	23 (48.9)	17 (34.0)		

Portable pedal exercise machine (PPEM); standing desk (SD). Values are mean (standard deviation) and 95% confidence intervals, unless specified. *Note:* Responses were reported on a 1 to 7 scale, where 1 = strongly disagree, 4 = neither agree or not, and 7 = strongly agree.
